# Vasospasm-Induced Takotsubo Cardiomyopathy: An Underrecognized Phenotype of Ischemia With Nonobstructive Coronary Arteries

**DOI:** 10.7759/cureus.108342

**Published:** 2026-05-06

**Authors:** Aaron Y Kim, Laura Bradel, Nimrah Hossain

**Affiliations:** 1 Internal Medicine, NewYork-Presbyterian Brooklyn Methodist Hospital, Brooklyn, USA; 2 Cardiology, NewYork-Presbyterian Brooklyn Methodist Hospital, Brooklyn, USA

**Keywords:** acetylcholine provocation testing, coronary artery vasospasm, ischemia with no obstructive coronary arteries (inoca), myocardial stunning, takotsubo cardiomyopathy (tcm)

## Abstract

Coronary vasospasm is a mechanistically distinct precipitant of takotsubo cardiomyopathy (TCM) that may produce transient left ventricular (LV) dysfunction through ischemia-induced myocardial stunning rather than adrenergic excess. Vasospasm-induced TCM remains underrecognized, and this case illustrates its distinct therapeutic implications, particularly in patients with ischemia with nonobstructive coronary arteries (INOCA). A 47-year-old perimenopausal woman with chronic tobacco use, hypertension, and a prior coronary angiogram performed approximately two years prior during a non-ST-elevation myocardial infarction workup demonstrating nonobstructive coronary artery disease presented with acute-onset substernal chest pressure, diaphoresis, and dizziness. Initial electrocardiography demonstrated anterior ST-segment depressions, and point-of-care echocardiography revealed apical ballooning with impaired LV systolic function. High-sensitivity troponin was mildly elevated at 53 ng/L. During hospitalization, the patient developed recurrent chest pain with new inferolateral ST-segment elevations, prompting emergent coronary angiography. Catheterization demonstrated diffuse multivessel vasospasm of the left anterior descending and left circumflex arteries with complete resolution following intracoronary nitroglycerin, consistent with functional rather than obstructive disease. Left ventriculography confirmed apical akinesis with basal hyperkinesis, and transthoracic echocardiography documented a left ventricular ejection fraction (LVEF) of 35%. LVEF recovered to 50-55% within 48 hours, consistent with the reversible myocardial stunning characteristic of TCM. This case satisfies the International Takotsubo Diagnostic Criteria for TCM and illustrates how diffuse epicardial vasospasm can fulfill the complete clinical syndrome of TCM in the absence of a catecholamine trigger. The patient’s history of recurrent nitrate-responsive angina with prior normal coronary imaging is consistent with a chronic vasospastic INOCA substrate that culminated in this acute event. In this case, calcium channel blockers and long-acting nitrates were selected to prevent smooth-muscle vasoconstriction, and beta-blockers were withheld, given the risk of paradoxical vasospasm worsening through unopposed alpha-adrenergic vasoconstriction. This case underscores the importance of mechanism-based phenotyping in TCM to guide appropriate, pathophysiology-directed therapy.

## Introduction

Takotsubo cardiomyopathy (TCM), also known as stress-induced cardiomyopathy or “broken heart syndrome,” is a form of transient left ventricular (LV) systolic dysfunction occurring in the absence of obstructive coronary artery disease [1]. It is classically precipitated by emotional or physical stress, leading to catecholamine-mediated myocardial stunning. TCM is named after the Japanese word “takotsubo” (“octopus pot”), reflecting the characteristic apical ballooning appearance during systole [2]. The proposed mechanism involves regional differences in adrenergic receptor density: the apex is enriched with β₂ receptors that, under extreme catecholamine excess, switch coupling from stimulatory Gs to inhibitory Gi proteins, suppressing apical contractility, while the β₁-rich base remains hypercontractile [3,4]. This apicobasal mismatch produces the classic ballooning pattern.

Although TCM was originally viewed as a purely neurohormonal disorder, growing evidence suggests that coronary vasomotor dysfunction, including both epicardial vasospasm and microvascular spasm, can produce similar transient wall-motion abnormalities. Coronary vasospasm involves reversible constriction of coronary vessels without fixed atherosclerotic obstruction, driven by endothelial dysfunction, reduced nitric oxide bioavailability, and Rho-kinase-mediated smooth muscle hypercontractility [5]. Cigarette smoking is among the most potent clinical triggers, impairing endothelial nitric oxide signaling and potentiating Rho-kinase activity [6,7]. When diffuse vasospasm involves major vessels such as the left anterior descending (LAD) or left circumflex (LCx) arteries, it can cause transient ischemia and a takotsubo-like LV dysfunction pattern [8].

This mechanism places vasospasm-induced TCM within the spectrum of ischemia with nonobstructive coronary arteries (INOCA), a clinically important entity in which patients experience angina or objective evidence of ischemia despite the absence of significant angiographic obstruction. Distinguishing vasospasm-induced from stress-induced TCM is critical, as the two require fundamentally different management approaches.

## Case presentation

A 47-year-old perimenopausal woman with a history of chronic tobacco use, hypertension, and a prior non-ST-elevation myocardial infarction (NSTEMI) workup that demonstrated angiographically normal coronaries, who denied any recent bereavement, acute psychological stressor, or significant life event preceding the presentation, presented with acute-onset substernal chest pressure (10/10) at 5:00 AM, accompanied by dizziness and diaphoresis. Initial electrocardiogram (ECG) demonstrated ST depressions in leads V1-V3. Symptoms transiently improved with sublingual nitroglycerin in the emergency department. High-sensitivity troponin on admission was 53 ng/L (mildly elevated above the 99th percentile upper reference limit (URL) for this assay). Bedside point-of-care ultrasound (POCUS) revealed mildly reduced LV systolic function with apical ballooning, raising concern for acute coronary syndrome versus TCM.

Despite initial stabilization, the patient developed recurrent crushing chest pain with worsening diaphoresis. Repeat ECG demonstrated new ST-segment elevations in leads II, III, aVF, V5, and V6, consistent with an acute inferolateral ST-elevation myocardial infarction (STEMI) pattern (Figure 1), and a STEMI code was activated.

**Figure 1:**
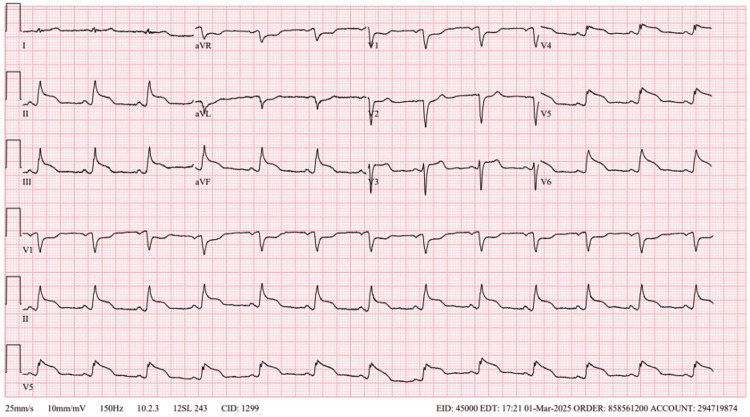
Electrocardiogram demonstrating ST-segment elevation in leads II, III, aVF, V5, and V6 consistent with acute inferolateral ST-elevation myocardial infarction pattern.

Urgent coronary angiography revealed nonobstructive coronary artery disease (CAD) with diffuse vasospasm of the LAD and LCx arteries, which resolved completely after intracoronary nitroglycerin (Figure 2). Left ventriculography demonstrated a severely reduced left ventricular ejection fraction (LVEF) with apical akinesis and basal hyperkinesis, consistent with TCM (Figure 3). Post-procedure transthoracic echocardiography (TTE) confirmed severe apical hypokinesis with an LVEF of 35% (Figure 4).

**Figure 2:**
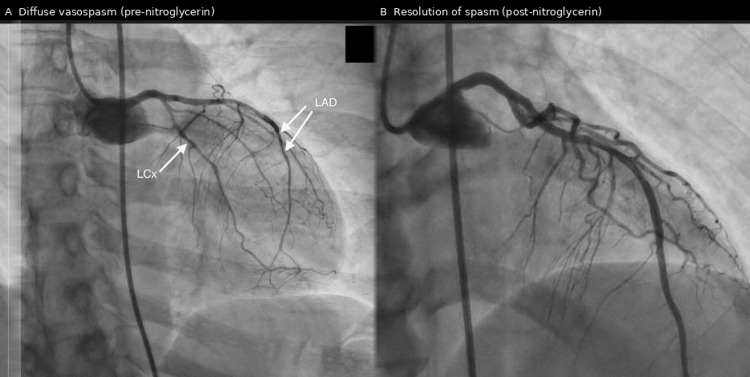
Coronary angiography of the left coronary system. (A) Diffuse vasospasm of the left anterior descending and left circumflex before intracoronary nitroglycerin administration. (B) Marked resolution of vasospasm following intracoronary nitroglycerin, confirming functional rather than obstructive disease. Note that matched RAO-CAUD cine-angiographic projections were not available; still images from the best available angiographic views are presented.

**Figure 3:**
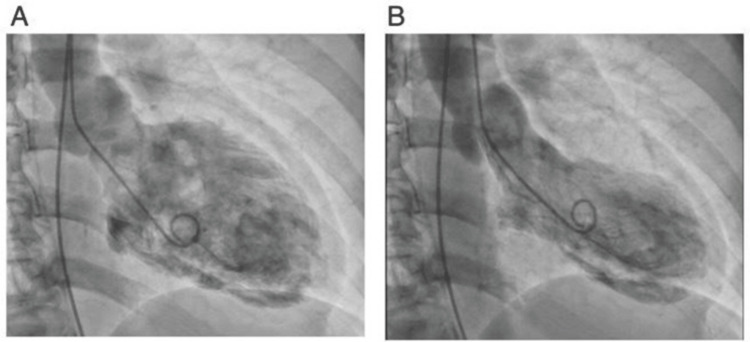
Left ventriculogram images showing (A) end diastole and (B) end systole, demonstrating apical ballooning and basal hyperkinesis consistent with the classic takotsubo pattern.

**Figure 4:**
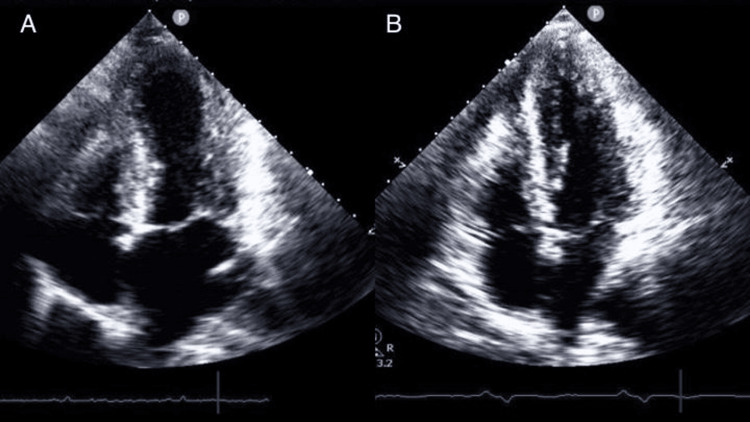
Transthoracic echocardiogram (apical four chamber view) showing (A) initial study with apical hypokinesis and basal hyperkinesis consistent with takotsubo cardiomyopathy, and (B) follow-up study demonstrating recovery of apical contractility.

The patient was initially managed with aspirin, clopidogrel, and short-term guideline-directed medical therapy with sacubitril/valsartan for heart failure with reduced ejection fraction, along with antianginal therapy comprising long-acting nitrates and diltiazem for vasospastic angina. Beta-blockers were withheld, given the vasospastic etiology. Repeat TTE at 48 hours demonstrated marked improvement in wall-motion abnormalities and recovery of LVEF to 50-55% (Figure 4B), at which point sacubitril/valsartan and clopidogrel were discontinued, given the rapid functional recovery. She was discharged on aspirin, diltiazem, and isosorbide mononitrate. At three-month outpatient follow-up, she remained symptom-free.

## Discussion

This case illustrates how diffuse epicardial vasospasm can trigger a takotsubo-like cardiomyopathy, producing the full clinical syndrome of chest pain, ST-segment changes, and transient LV dysfunction in the absence of fixed coronary obstruction. The directly visualized multivessel vasospasm, confirmed by associated wall-motion abnormality and STEMI-pattern ECG changes and reversible with intracoronary nitroglycerin, provides strong mechanistic evidence for vasospasm as the precipitant. In this patient, chronic tobacco use likely served as the key predisposing factor, consistent with her history of nitrate-responsive anginal episodes and prior angiographically normal coronaries.

Applying the International Takotsubo Diagnostic Criteria

This case satisfies the International Takotsubo (InterTAK) Diagnostic Criteria for TCM [9]: (1) transient LV wall-motion abnormality with apical ballooning exceeding a single epicardial territory; (2) absence of obstructive CAD on angiography; (3) new ECG abnormalities (ST-segment elevation followed by T-wave changes); (4) troponin mildly but not markedly elevated, disproportionately to the extent of wall-motion dysfunction; (5) no evidence of pheochromocytoma or myocarditis; and (6) documentation of LV wall-motion recovery, as demonstrated by return of LVEF to 50-55% within 48 hours. Importantly, the InterTAK criteria do not require an emotional or physical stressor, recognizing that TCM can arise via non-catecholamine mechanisms, as in this vasospasm-driven case.

Demographic and hormonal context

TCM demonstrates a striking female predominance, affecting women in more than 90% of reported cases, with postmenopausal and perimenopausal women at highest risk [1,9]. Estrogen plays a key protective role in coronary vasomotor regulation: it promotes endothelial nitric oxide synthase activity, enhances nitric oxide bioavailability, and attenuates adrenergic vascular reactivity. The decline in estrogen during the perimenopausal transition renders the coronary endothelium more susceptible to vasomotor instability, both through impaired vasodilatory reserve and heightened sensitivity to adrenergic and vasoconstrictive stimuli [10]. This patient, a 47-year-old perimenopausal woman with chronic tobacco use, represents the convergence of hormonal and environmental risk factors that characterize the highest-risk phenotype for vasospasm-mediated coronary events.

Mechanistic basis of vasospasm-induced takotsubo cardiomyopathy

In vasospasm-induced TCM, apical dysfunction arises through a distinct but convergent mechanism. Rather than a systemic catecholamine surge, transient but severe coronary constriction produces brief, profound ischemia: depletion of adenosine triphosphate, intracellular calcium accumulation, and free radical generation transiently halt myocardial contraction while sparing cellular integrity [11]. When coronary flow is restored, whether spontaneously or after nitrate administration, metabolic homeostasis recovers and contractile function returns within hours to days, as seen in this patient’s 48-hour LVEF recovery. Histopathologic studies of vasospasm-related TCM demonstrate interstitial edema and contraction band necrosis without significant myocyte loss, distinguishing this entity from true myocardial infarction [12].

This patient’s presentation also fits within the broader INOCA spectrum. INOCA encompasses pathophysiologic mechanisms, including epicardial vasospasm, microvascular dysfunction, and endothelial dysregulation, that cause myocardial ischemia despite angiographically unobstructed arteries [13]. This patient represents the severe end of the INOCA continuum, where diffuse epicardial vasospasm produced sufficient ischemia to cause mechanical stunning and systolic failure. Her recurrent nitrate-responsive episodes and prior normal coronary studies are consistent with a chronic vasospastic INOCA substrate that eventually precipitated this acute event. It should be noted that this presentation also fulfills the criteria for myocardial infarction with nonobstructive coronary arteries (MINOCA), which provides a complementary acute mechanistic framework: within the MINOCA construct, vasospasm is a recognized precipitant of troponin elevation and wall-motion abnormality in the absence of obstructive coronary disease.

Diagnosis and management

Differentiating vasospasm-induced from stress-induced TCM has direct therapeutic consequences. In vasospasm-driven disease, calcium channel blockers (diltiazem or verapamil) are first-line to prevent smooth-muscle vasoconstriction, and long-acting nitrates such as isosorbide mononitrate maintain vasodilation and reduce recurrence. Aggressive smoking cessation counseling is equally essential, as continued nicotine exposure perpetuates endothelial injury and Rho-kinase activation. In contrast, beta-blockers are the mainstay for stress-induced TCM, where blunting the sympathoadrenergic surge is the therapeutic goal. Misclassifying a vasospasm variant as stress-induced TCM risks treating with beta-blockers, which may paradoxically worsen spasm through unopposed alpha-adrenergic vasoconstriction [14].

One nuance in this patient’s discharge regimen warrants comment: high-dose aspirin can theoretically inhibit prostacyclin-mediated vasodilation and potentially exacerbate vasospasm. While low-dose aspirin was retained given her prior NSTEMI history, this consideration should be weighed carefully in patients without a clear atherosclerotic indication.

In this case, directly visualized diffuse vasospasm with complete resolution after intracoronary nitroglycerin was sufficient to confirm the diagnosis without additional provocative testing. Spontaneous angiographic spasm, as demonstrated here, provides more definitive mechanistic evidence than pharmacologic provocation. However, in patients with recurrent chest pain, nonobstructive coronaries, and a TCM-pattern LV dysfunction without directly visualized spasm, intracoronary provocation testing per European Association of Percutaneous Cardiovascular Interventions and Coronary Vasomotion Disorders International Study guidance is essential to avoid misclassification [13]. A positive test is defined as 90% or greater luminal narrowing with ischemic ECG changes for epicardial spasm, or ischemic ECG changes without visual narrowing for microvascular spasm [10].

## Conclusions

This case highlights vasospasm-induced TCM as a rare but clinically important mimic of acute coronary syndrome, presenting with chest pain, ST-segment changes, and troponin elevation in the setting of nonobstructive coronaries, particularly in patients with active tobacco use and a history of nitrate-responsive angina. Unlike conventional stress-induced TCM, which is driven by catecholamine excess and managed with beta-blockers, vasospasm-induced TCM results from transient coronary vasoconstriction and requires calcium channel blockers and long-acting nitrates. Misclassification carries real clinical risk, including recurrent ischemia from inappropriate beta-blocker use. This case underscores the need for heightened awareness of coronary vasomotor disorders within the INOCA spectrum and the value of mechanism-specific therapy in optimizing long-term outcomes.
